# Evaluation of a Luminometric Cell Counting System in Context of Antimicrobial Photodynamic Inactivation

**DOI:** 10.3390/microorganisms10050950

**Published:** 2022-04-30

**Authors:** Moritz Lehnig, Sarah Glass, Norman Lippmann, Svitlana Ziganshyna, Volker Eulenburg, Robert Werdehausen

**Affiliations:** 1 Department of Anaesthesiology and Intensive Care, Medical Faculty, University of Leipzig, 04103 Leipzig, Germany; moritz.lehnig@medizin.uni-leipzig.de (M.L.); svitlana.ziganshyna@medizin.uni-leipzig.de (S.Z.); 2 Leibniz Institute of Surface Engineering (IOM), 04318 Leipzig, Germany; sarah.glass@hereon.de; 3 Institute for Medical Microbiology and Epidemiology of Infectious Diseases, University of Leipzig, 04103 Leipzig, Germany; norman.lippmann@medizin.uni-leipzig.de

**Keywords:** photodynamic therapy, photodynamic inactivation, luminescence measurement, antimicrobial resistance, TMPyP, THPTS

## Abstract

Antimicrobial resistance belongs to the most demanding medical challenges, and antimicrobial photodynamic inactivation (aPDI) is considered a promising alternative to classical antibiotics. However, the pharmacologic characterization of novel compounds suitable for aPDI is a tedious and time-consuming task that usually requires preparation of bacterial cultures and counting of bacterial colonies. In this study, we established and utilized a luminescence-based microbial cell viability assay to analyze the aPDI effects of two porphyrin-based photosensitizers (TMPyP and THPTS) on several bacterial strains with antimicrobial resistance. We demonstrate that after adaptation of the protocol and initial calibration to every specific bacterial strain and photosensitizer, the luminometric method can be used to reliably quantify aPDI effects in most of the analyzed bacterial strains. The interference of photosensitizers with the luminometric readout and the bioluminescence of some bacterial strains were identified as possible confounders. Using this method, we could confirm the susceptibility of several bacterial strains to photodynamic treatment, including extensively drug-resistant pathogens (XDR). In contrast to the conventional culture-based determination of bacterial density, the luminometric assay allowed for a much more time-effective analysis of various treatment conditions. We recommend this luminometric method for high-throughput tasks requiring measurements of bacterial viability in the context of photodynamic treatment approaches.

## 1. Introduction

The increasing occurrence of antibiotic resistance in bacteria is a major clinical challenge. Despite intense research, only very few new antibiotics with a novel mechanism of action have been identified [1,2]. Therefore, new strategies that do not depend on specific bacterial structures, such as antimicrobial photodynamic inactivation (aPDI), constitute highly interesting alternatives [3,4,5]. In PDI, a non-toxic photosensitizer binds to or is taken up by the bacteria [6]. For aPDI, porphyrin-based sensitizers are currently evaluated most often [5], but there is a broad range of other naturally occurring as well as synthetic compounds with photosensitizing properties [3,7], including quinone derivatives, phenothiazines such as methylene blue, and xanthenes, including phthaleins and fluorescein derivatives such as *Rose Bengal*. Upon illumination, these sensitizers induce the generation of reactive oxygen species (ROS), such as singlet oxygen [8,9], that can inactivate bacteria in a variety of ways [10,11] and independently of possible antibiotic resistances [7].

Ongoing research focuses on further optimization of photosensitizers for specific applications [12,13], as well as on the in-detail characterization and comparison of different photosensitizers in vitro [14,15,16]. Additionally, photosensitizers were tested in in vivo models for critical infections [17,18,19]. Unfortunately, the assessment of one central readout in these experiments, the number of viable bacteria, is labor-intensive and time-consuming, which limits the amount of processable samples. Therefore, a scalable approach that allows for the parallel analysis of various treatment conditions and a great variety of different pathogens that are clinically relevant is urgently required. 

In this study, we aimed to establish a reliable, fast and scalable method for the detection of viable bacteria with a faster processing time and limited manual manipulation, as compared to standard processes that involve agar plate cultures and the counting of bacterial colonies formed after incubation [20,21]. Previously described methods with higher throughput, such as the antibody-based Raman technique for multiplex optical quantification, do require sophisticated and expensive equipment [22], and PCR methods cannot discriminate between inactivated and viable bacteria [23]. Thus, we used a chemiluminescence-based cell viability system that enables the detection of ATP-dependent luminescence in a 96-well format as a surrogate for the number of viable bacteria in solution. This system is based on firefly luciferin, which is catalyzed by recombinant luciferase in the presence of magnesium (Mg^2+^) ions, ATP and molecular oxygen. This mono-oxygenation of luciferin is a chemically exothermic reaction that results in oxyluciferin, adenosine monophosphate (AMP), inorganic pyrophosphate, carbon dioxide (CO_2_) and yellow-green (550–570 nm) light emission [24]. Therefore, the amount of ATP contained in the sample to be analyzed can be inferred from the amount of light measured [25].

For the evaluation of the modified luminescence-based method, we selected the porphyrin-based photosensitizers TMPyP and THPTS, which are both cationic and water-soluble [5,13] but differ in their optical properties and require different light activation. 

We investigated the photodynamic inactivation of several bacterial strains, including Gram-positive *Staphylococcus aureus* and *Enterococcus faecium* and Gram-negative bacteria such as *Escherichia coli*, *Klebsiella pneumoniae*, *Actinetobacter baumanii*, *Pseudomonas aeruginosa*, *Achromobacter xylosoxidans* and *Morganella morganii*. For all of these pathogens, antimicrobial resistances (AMR) have been described that are a serious threat and a great challenge to the global public health today [1].

## 2. Materials and Methods

### 2.1. Bacterial Strains and Growth Media

All investigated bacterial strains are listed in Table 1. Individual patient-derived strains were identified by MALDI-TOF mass spectrometry (VITEK-MS, bioMérieux, Marcy-l’Étoile, France) in accordance to Tanis et al. [26]. Antibiotic resistances were tested according to the ISO 20776-1 microbroth dilution method.

Blood agar containing Columbia Blood Agar Base (Oxid, Wesel, Germany) with 5% sheep blood (Fiebig, Idstein-Niederauroff, Germany), 2xYT (Trypton, Yeast, NaCl) and brain–heart infusion (BHI; Oxid) dissolved in 0.9% NaCl solution were used as growth media.

### 2.2. Culture Conditions

The bacteria were cultured at 37 °C and 5% CO_2_ on blood agar plates overnight. Individual colonies were diluted in 0.9% NaCl solution to an initial bacterial density that was optically equivalent to McFarland standard no. 1, corresponding to a concentration of approx. 3 × 10^8^ colony-forming units (CFU)/mL. The resuspended bacteria were diluted to a final concentration of 7.5 × 10^7^ CFU/mL in 0.9% NaCl solution containing 50% BHI and a photosensitizer at the indicated concentrations. 

For the detection of viable bacteria by luminescence measurement, PDI-treated bacterial suspensions were incubated in the 24-well plate used for PDI at 37 °C and 5% CO_2_ for 90 min and stored at −80 °C (<12 h) until detection.

### 2.3. Assessment of Bacterial Viability

Quantification of ATP was performed by commercially available luciferase-based assay system (BacTiter-Glo™ Microbial Cell Viability Assay, Promega, Madison, WI, USA). Light signals were detected by a luminescence microplate reader (Berthold Technologies, Centro LB 960, Bad Wildbad, Germany) in non-transparent white 96-microwell plates (Thermo Scientific, Waltham, MI, USA) and analyzed by Microwin 2010 software (version 5.17, Mikrotek Laborsysteme GmbH, Neunkirchen-Seelscheid, Germany). Samples (50 µL) were measured in duplicates with a registration time of 1 s per well. Luminescence data are presented as raw RLU data detected after incubation without any adaptation.

For classic clonogenic bacterial density quantification immediately after aPDI, 100 µL of bacterial suspension was diluted by a factor of 10^−3^ to 10^−6^ in 0.9% NaCl solution in order to achieve a high counting accuracy, then spread out with a Drigalski spatula on blood agar plates and incubated for 20 h ± 4 h at 37 °C and 5% CO_2_. Subsequently, the agar plates were photographed, and the colony numbers were counted using Image J (Rasband, W.S., U.S. National Institutes of Health, Bethesda, MD, USA) to determine the colony-forming units (CFU). In contrast to other bacterial strains, *Achromobacter xylosoxidans* required 48 h of incubation at 37 °C and 5% CO_2_ in order to ensure sufficient visibility of colonies.

### 2.4. Photodynamic Treatment

The photosensitizer TMPyP 4,4′,4″,4″′-(5,10,15,20-tetrayl)tetrakis(1-methylpyridin-1-ium)porphyrin tetratosylate was purchased in the highest available purity (97%) (Product 323497, Sigma Aldrich Chemistry GmbH, Steinheim, Germany). The photosensitizer THPTS (3,3′,3″,3″′-(7,8,17,18-tetrahydro-21H,23H-porphyrine-5,10,15,20-tetrayl) tetrakis[1-methyl-pyridinium] tetratosylate, purity 95%), characterized in other studies [27,28], was purchased from TetraPDT GmbH (Rackwitz, Germany).

Illumination was performed in transparent 24-well plates (Cellstar, Greiner bio-one, Frickenhausen, Germany). For TMPyP, we used a custom-made planar LED array-based illumination device, previously analyzed by Habermann et al. [29], with an intensity maximum of 13 mW/cm^2^ at λ_cent_ = 420 nm at a distance of 8 cm. This setup enabled a homogenous light application of all samples on the microwell plate.

For THPTS illumination, a custom-made planar LED array (LEDs: SMB1N 760d, Roitner Laser, Vienna, Austria) with an intensity maximum of 18 mW/cm^2^ at λ_cent_ = 760 nm at a distance of 8 cm was used. A power supply (Bio Rad, Power Pac HC, Hercules, CA, USA) was used to power this light source. Light intensity was measured with a power meter (FieldMax2 TP, Coherent, Portland, OR, USA).

Both photosensitizers were prepared and stored in stock solutions (0.8 mM in 0.9% NaCl) in the dark at 4 °C for a maximum of three weeks before use. For photodynamic treatment, the photosensitizer stock solution was added to the bacterial suspensions to obtain the final concentrations indicated (0 to 200 µM). aPDI was performed at a volume of 1 mL per well.

After the addition of the photosensitizer, all samples were preincubated for 20 min. For TMPyP-treated samples, illumination was started for 0, 60, 90 and 120 min, as previously described [30,31]. For THPTS-based aPDI treatment, bacteria were illuminated for 0, 4, 12, 36 and 108 min in accordance with Ziganshyna et al. [32]. Additional samples were stored under identical conditions but protected from light (dark control). After light exposition, the samples were protected from light and subjected to luminometric cell counting or culture-based determination of bacterial density.

**Table 1:** Classification of bacterial strains by Gram-stain and antibiotic resistance.

Classification	Gram-Stain	Resistances [33]
*Escherichia coli*	negative	Extended spectrum beta-lactamase (ESBL)
*Staphylococcus aureus*	positive	Methicillin-resistant (MRSA)
*Klebsiella pneumoniae*	negative	ESBL-producing, fluoroquinolone-resistant, carbapenem-resistant (CRE)
*Acinetobacter baumanii*	negative	Extensively drug-resistant (XDR)
*Enterococcus faecium*	positive	Vancomycin-resistant (VRE)
*Pseudomonas aeruginosa*	negative	Multidrug-resistant (MDR) (including carbapenems)
*Morganella morganii*	negative	-
*Achromobacter xylosoxidans*	negative	Extensively drug-resistant (XDR)

### 2.5. Spectrometry

The absorption spectrometry of photosensitizers and bacterial growth media, as well as the luminescence spectrometry of the Cell Viability Assay, was performed with a SpectraMax M5 instrument (Molecular Devices, San José, CA, USA).

### 2.6. Statistical Analysis

Data are presented as mean ± SD. Differences between groups were determined using one- or two-way ANOVA and Bonferroni post hoc test. For cross-correlation analysis, corresponding value pairs determined by the two different detection methods were plotted against each other to assess a linear correlation. Calculations were performed with GraphPad Prism (version 7.0, GraphPad, San Diego, CA, USA).

## 3. Results

### 3.1. Evaluation of a Luminescence-Based Approach for the Determination of aPDI Effects

**Figure 1:**
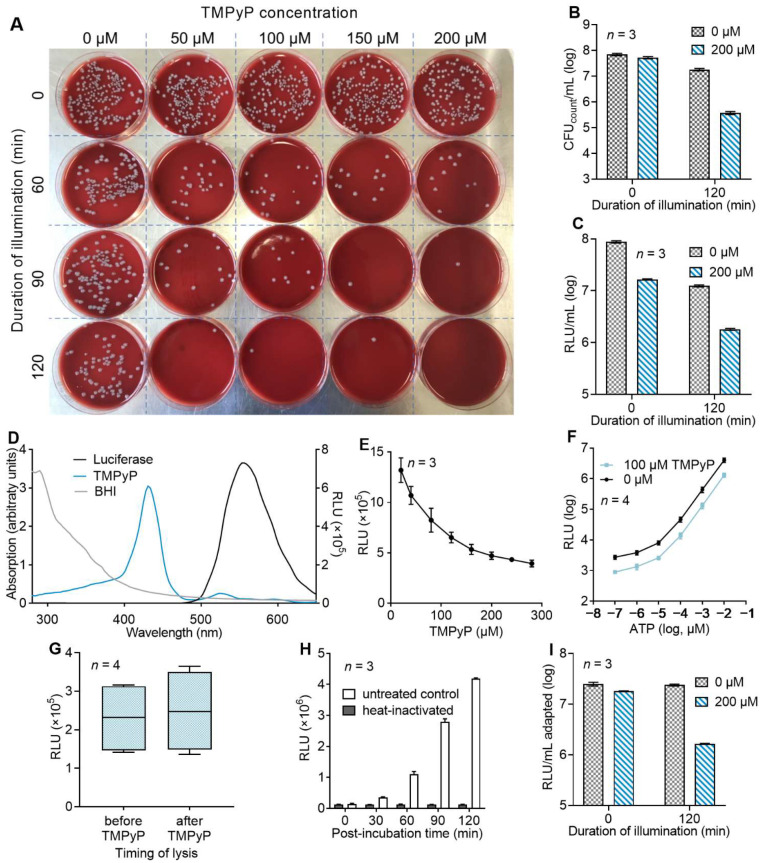
ATP-based luminometry as a novel approach to measure aPDI effects in vitro. (**A**) *E. coli* bacteria were treated with TMPyP at the indicated concentration and exposed to light (13 mW/cm^2^ at λ_cent_ = 420 nm; 0.78 J/cm^2^ per minute) for the indicated durations. Bacteria were spread on blood agar plates (dilution factor 10^−5^) and incubated for 20 h (37 °C, 5% CO_2_). (**B**) aPDI effect of the experiment described in A without or with 200 µM TMPyP determined as CFU_count_/mL. (**C**) *E. coli* were incubated without or with 200 µM TMPyP and treated for 0 or 120 min with light as described in A. Bacterial viability was determined by ATP-induced luminescence in bacterial lysates. (**D**) Absorption spectrum of 40 µM TMPyP dissolved in 50% BHI bacterial growth medium dissolved in 0.9% NaCl solution. Additionally, the luminescence spectrum of the luciferase used in the cell viability assay and absorption spectrum of the BHI medium are displayed. (**E**) Effect of different TMPyP concentration on ATP-induced luminescence; TMPyP at indicated concentrations was supplemented with 5 nM ATP (in 0.9% NaCl solution) and ATP-induced luminescence was determined (*n* = 3). (**F**) Luminescence elicited by ATP at the indicated concentrations (in 0.9% NaCl) in the presence or absence of 100 µM TMPyP (*n* = 4). (**G**) Effect of TMPyP on bacterial ATP metabolism. *E. coli* suspended in 50% BHI were lysed before or after addition of 200 µM TMPyP (*n* = 4). (**H**) *E. coli* suspended in 50% BHI broad medium were heat-inactivated or left untreated and incubated at 37 °C, 5% CO_2_ in the same solution as active bacteria for the indicated duration. Bacteria were lysed, and luminescence was measured with the cell viability assay (*n* = 3). (**I**) aPDI effect of the experiment described in C with 200 µM TMPyP, measured 90 min after incubation (37 °C, 5% CO_2_) and adjusted to highest PS concentration (200 µM) in all samples prior to measurements.

To test whether a luminometric quantification of ATP in bacterial lysates after aPDI treatment was suitable for the determination of the aPDI effects on bacterial growth, we determined the effect of 0 to 200 µM TMPyP and illumination for 0 to 120 min with 13 mW/cm^2^ at λ_cent_ 420 nm on *E. coli* using conventional bacterial culture and colony counting. Consistent with previous results, we found a reduction in the bacterial colony number [34], which depended on the photosensitizer concentration and illumination time (Figure 1A,B).

When we analyzed the bacterial viability as determined by the ATP concentration in bacterial cell lysates after aPDI treatment following the manufacturer’s instructions, we observed a clear reduction in the luminescence in bacterial lysates after aPDI treatment, as compared to lysates that were only incubated with the photosensitizer but that were protected from light, suggesting that the aPDI effects can be determined using this approach. However, the addition of only the photosensitizer (without any illumination) also caused a significant reduction in detected luminescence, suggesting that the photosensitizer interferes with the assay system (Figure 1C). Therefore, we decided to develop an adapted protocol by performing additional preliminary experiments.

To determine whether the optical properties of the photosensitizers interfere with the luminescence measurement, we determined the absorption spectrum of TMPyP dissolved in 50% BHI-NaCl solution. Consistent with the optimal wavelength for aPDI treatment, the absorption maximum was detected at λ = 428 nm. The absorption of the 50% BHI medium itself continually decreases at wavelengths higher than 320 nm. At λ = 428 nm, the absorption declines below 0.52 arbitrary units. To determine possible interference with the luminescence detection assay system, the emission spectrum of the used luciferase was determined. Here, the highest intensity of the emitted light was detected at λ ≈ 556 nm, with significant intensities being observed in the range from 480 nm to 730 nm. These data indicated that there is no overlap between the main absorption maximum of TMPyP and the assay-induced luminescence. Nevertheless, there was an increased optical density of the solution from λ = 480 to 730 nm (Figure 1D). To test whether TMPyP interferes with the detection of luciferase-emitted luminescence, we determined the ATP-induced luminescence in our assay system in the presence of increasing concentrations of TMPyP. Indeed, a dose-dependent reduction in the apparent luminescence was observed (Figure 1E). To test whether this was due to the optical properties of TMPyP or due to the direct interference of TMPyP with the luciferase or any other component of the detection system, we determined the luminescence elicited by different ATP concentrations in the presence or absence of TMPyP. Here, in all samples, a constantly decreased luminescence by 67.9% ± 3.3% (*n* = 4) in the presence of TMPyP was observed (Figure 1F), demonstrating that TMPyP functions as a quencher for luciferase-emitted luminescence without directly interfering with the detection system. To compensate for this quenching, in all subsequent experiments, the TMPyP concentration was adjusted to the highest concentration used in the respective experiment prior to luminescence measurements.

Next, we investigated whether TMPyP itself had any direct influence on the ATP metabolism of the bacteria or any effect on the lysis efficacy prior to the luminescence detection. Therefore, we performed the bacterial lysis of Gram-negative *Escherichia coli* before and after TMPyP addition and compared the assay-induced luminescence. At a concentration of 200 µM TMPyP, representing the highest used concentration during photodynamic treatment, no relevant influence on the luminescence of the assay and the associated ATP release was observed (Figure 1G).

Since ATP content is used for the luminometric detection of viable bacteria, which is dependent on bacterial metabolism, we assumed that the time between aPDI treatment and bacterial lysis was a critical variable. Here, a comparison of the luminescence elicited by lysates from heat-inactivated *E. coli* to that from viable bacteria revealed a time-dependent increase in the difference in luminescence compared to the control group (Figure 1H). Similar findings resulted from experiments with extensively drug-resistant (XDR) *Achromobacter xylosoxidans*, Vancomycin-resistant Enterobacteriaceae (VRE) *Enterococcus faecium*, multidrug-resistant (MDR) *Pseudomonas aeruginosa* and XDR *Acinetobacter baumanii* (Figure S1).

Another source of error to be considered was the background luminescence of the luciferase-based assay, which is influenced by the type of bacterial growth medium because of the luminescence-inducing ATP in their ingredients. Regarding our aPDI experiments, two growth media were compared. A 17.5 times lower background luminescence of 4436 ± 88.6 RLU was observed in the BHI bacterial growth medium compared to 775,434 ± 1399.6 RLU in the 2xYT growth medium (Figure S2A).

Based on these findings, we implemented the following adjustments to the protocol for luminescence-based determination: First, the TMPyP concentration of all bacterial lysates was adjusted to the highest concentration in the respective experiment. Second, all samples were incubated for 90 min after the aPDI treatment before luminescence detection. With these optimizations, we again determined the aPDI effect caused by 200 µM TMPyP on *E. coli* in comparison to samples that were only illuminated without TMPyP (light control) or only treated with the photosensitizer without illumination (dark control) (Figure 1I). The relative reduction in luminescence in the dark control by 26.5% ± 5.2% were now similar to the relative reduction observed with the conventional colony-counting method (by 24.2 ± 7.5%; Figure 1B). Moreover, a significant aPDI effect was detected by the reduction in luminescence (by 91.0% ± 0.2%, Figure 1I) and by the reduction in the counted colony number by 99.2% ± 0.1% (Figure 1B).

### 3.2. Correlation of Results for Density of Viable Bacteria Determined by Luminescence or Conventional Colony Counting

To allow for a direct comparison of the ATP concentration within the lysates as determined by the luminometric measurement with the number of bacteria found in a given sample, we developed a standardized protocol that allowed for the conversion of the detected luminescence (RLU) to the corresponding and more conventional measuring unit CFU/mL.

**Figure 2:**
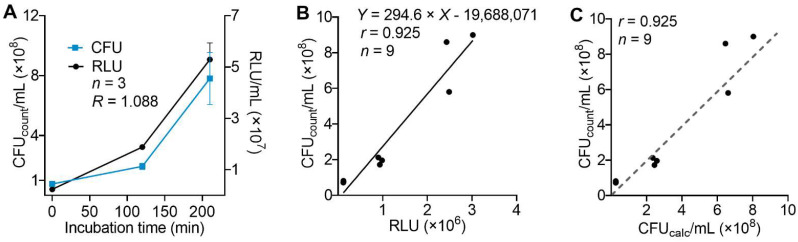
Correlation of luminescence-based readout and bacterial density as determined by colony counting. (**A**) *E. coli* suspended in 50% BHI broad medium were incubated at 37 °C, 5% CO_2_, and CFU_count_/mL were determined by spreading aliquots on agar plates and counting bacterial colonies after 20 h. In parallel, bacterial density was determined on the basis of ATP-dependent luminescence in bacterial lysates. The bacterial growth rate (R) was defined as the logarithm of RLU_210_ to the base of RLU_120_, assuming exponential growth (similar to that obtained on basis of CFU_count_). (**B**) Linear correlation between luminescence values as relative luminescence units (RLU) and bacterial density determined as CFU_count_/mL. Luminescence detected in bacterial lysates from samples heat-inactivated at *t* = 0 min were set to zero CFU/mL. (**C**) Comparability of recalculated bacterial density (CFU_calc_/mL) to manually counted bacterial density (CFU_count_/mL) of ESBL *E. coli*.

To this end, the corresponding values from both detection types of *E. coli* were compared at three different incubation times (*t* = 0, 120, 210 min) and, therefore, at increasing densities. This enabled us to determine the bacterial growth rate (R) and the resulting ATP concentration in a bacterial solution under these conditions (Figure 2A). The bacterial growth rate (R) was defined as the logarithm of the *RLU*_210 min_ value to the base of the *RLU*_120 min_ value, assuming exponential growth. Additionally, we assumed that the luminescence detected in the lysates of heat-inactivated bacteria equaled a bacterial density of zero.

To calculate bacterial density in aPDI experiments after incubation, the detected raw RLU data had to be adapted before further calculation, accounting for the bacterial growth rate (R): $RLU_{adapt}={\left(RLU_{PDI}\right)}^{1/R}$.

The corresponding data from both detection methods were then used for linear regression analysis (Figure 2B), which allowed us to convert RLU data to calculated CFU_calc_/mL with a good correlation for TMPyP-based aPDI and *E. coli* bacteria (*r* = 0.925, Figure 2C).

### 3.3. Standard Protocol to Establish Luminescence-Based Cell Density Determination for aPDI Experiments with Other Photosensitizers and Bacterial Strains

**Figure 3:**
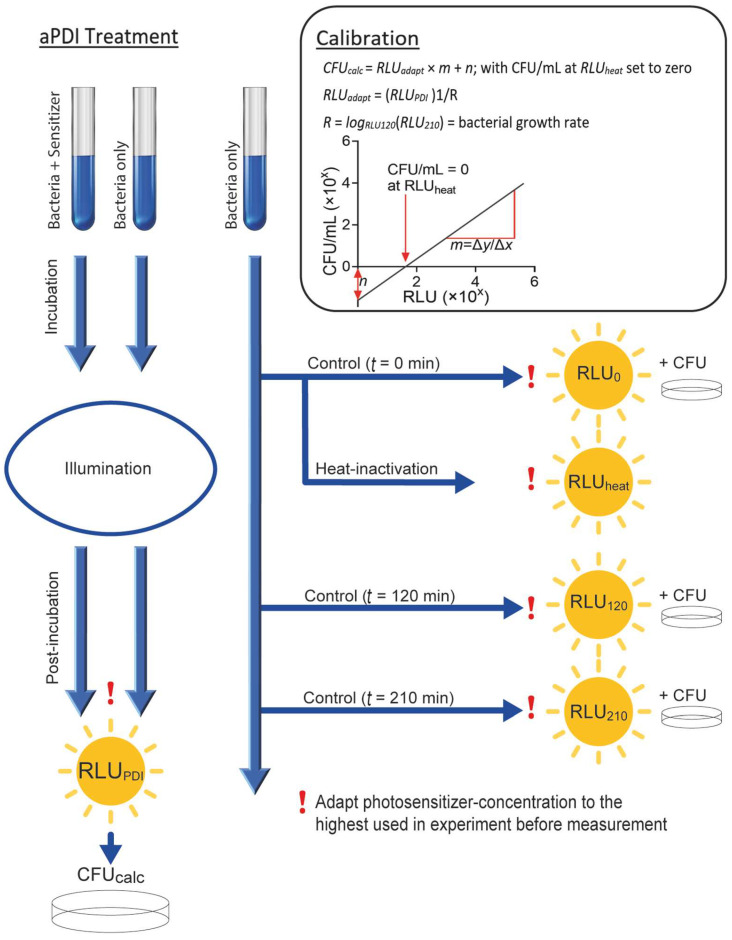
A schematic presentation of the standard procedure for determining aPDI effects with a given photosensitizer on any bacterial strain. The required calibrations to correlate the luminescence reading to previously determined colony-forming units (CFU) are illustrated. In order to calculate bacterial density (CFU_calc_) from luminescence values, a regression analysis of corresponding data pairs from control group experiments is required. Corresponding values before illumination, after complete illumination and after post-incubation enable a linear regression analysis between luminescence and bacterial density. Additionally, the bacteria-specific replication time (*R*) was determined by this measurement and used to calculate the bacterial density after aPDI. The luminescence observed in samples from heat-inactivated bacteria was defined as the luminescence emitted at a density of zero CFU/mL for viable bacteria. Process of calculation after calibration: 1. Determine bacterial growth rate (*R*) = $\log_{RLU\left(120\min\right)}RLU\left(210\min\right)$; 2. Calculate bacterial density in RLU (*RLU adapt*) from luminescence data after incubation (*RLU adapt* = *RLU_PDI_*^1/R^); 3. Use RLU_adapt_ to calculate CFU_calc_/mL by linear regression, set to zero CFU/mL at luminescence of heat-inactivated bacteria according to the formula CFU_calc_/mL = RLU_adapt_/mL × *m* + *n* with *m* and *n* derived from linear regression-fitting results; 4. Define calculated data less than or equal to zero as zero CFU/mL.

Taken together, we propose the following steps to determine calibration factors when performing aPDI experiments with new bacterial strains and/or other aPDI conditions, such as, for example, a novel photosensitizer. Our aPDI treatment protocol (Figure 3) consists of 20 min preincubation of samples with the photosensitizer, followed by illumination as described in the respective aPDI protocol. The post-aPDI incubation period was standardized to 90 min. Control samples without PS and illumination are mandatory. As described above, the photosensitizer concentration in all samples must be adjusted to the maximum concentration used in the experiment prior to luminometric measurements.

The correlation factors between luminescence intensity (in RLU) and bacterial density (in CFU_count_/mL) are determined by analyzing the untreated bacterial samples (*n* = 3) using both methods after incubations times of *t* = 0, 120, 210 min (corresponding to the incubation times used in the aPDI experiments). Additionally, heat-inactivated samples of the control (*t* = 0 min) are measured in order to determine the luminescence that is detected at a bacterial density of zero. The bacterial growth rate (R) was determined between 120 min and 210 min (corresponding to 90 min post-incubation). This enables a calculation of the bacterial density data from the luminescence data directly after treatment (120 min), using the luminescence data detected after incubation (210 min).

The observed comparability between luminescence intensity (RLU) and bacterial density (CFU_count_/mL) in the controls (*n* = 3) then allows for the calculation of bacterial density (CFU_calc_/mL) from aPDI data, according to the following equation (and Figure 3):

$$\begin{array}{l} R=\log_{RLU\left(120\min\right)}RLU\left(210\min\right)=bacterial\;growth\;rate\\ RLU_{adapt}={\left(RLU_{PDI}\right)}^{1/R}\;\\ CFU_{calc}=RLU_{adapt}\times m+n \end{array}$$

### 3.4. Evaluation of the Luminescence-Based Assay Protocol with Different Bacterial Strains in the Context of aPDI

**Figure 4:**
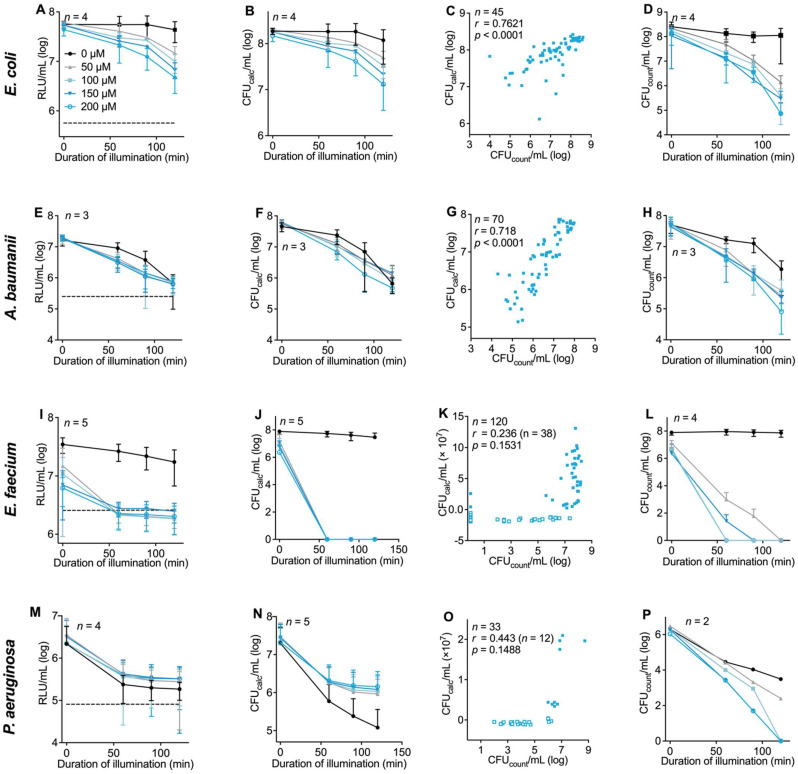
Evaluation of aPDI effects of TMPyP on different bacterial strain as determined by ATP-evoked luminescence and manual counting of bacteria colonies. Data from the aPDI experiments are presented. TMPyP at indicated concentrations was illuminated with 13 mW/cm^2^ resulting in 0 J/cm^2^ (0 min), 46.8 J/cm^2^ (60 min), 70.2 J/cm^2^ (90 min), 93.6 J/cm^2^ (120 min) at λ_cent_ = 420 nm; the dashed line shows the luminescence of heat-inactivated bacteria. (**A**) ATP-induced luminescence of ESBL *E. coli* after aPDI. (**B**) Calculated bacterial density (CFU_calc_) of ESBL *E. coli* based on luminometric measurements. (**C**) Correlation between CFU_calc_ and manually determined bacterial density (CFU_count_) in ESBL *E. coli*; *r* = correlation coefficient. (**D**) CFU_count_ of viable ESBL *E. coli* after aPDI. (**E**–**H**) Results for XDR *A. baumanii* as described above for *E. coli*. (**I**–**L**) Results for VRE *E. faecium*; calculated values less the zero were excluded per definition (empty boxes). (**M**–**P**) Results for MDR *P. aeruginosa*.

Subsequently, the above-established protocol was used to analyze the aPDI effects of TMPyP on different clinically relevant patient-derived bacterial strains. To obtain the required bacterial growth rate and conversion factors for the individual bacterial strains used, samples were prepared and analyzed in accordance with Section 2.3 and Figure 3. For most bacterial strains tested in this study, the protocol led to a good correlation between luminometric registrations and manually counted colonies. Here, in the Gram-negative extended spectrum beta-lactamase (ESBL) *Escherichia coli*, which were used to establish the protocol, we found an aPDI-induced RLU decrease of 89.6% ± 3.1% with 200 µM TMPyP and an illumination time of 120 min (Figure 4A). This corresponds to a calculated reduction in bacterial density (CFU_calc_) of 95.5% ± 4.8% (Figure 4B). Conventional colony counting resulted in a reduction in bacterial density of 99.9% ± 0.1% (Figure 4D) after aPDI treatment. A correlation analysis between the two methods for bacterial density measurement revealed a correlation coefficient of *r* = 0.762 (*p* < 0.0001, Figure 4C). This result indicates that the aPDI effects on *E. coli* are slightly underestimated when assessed with the luminescence method.

To extend our experiments to other Gram-negative bacterial strains not previously analyzed, we analyzed the effect of TMPyP-elicited aPDI effects on XDR *Acinetobacter baumanii*. Here, we observed a blue-light susceptibility that was independent from TMPyP, which caused a reduction in luminescence after 120 min illumination of 96.0 ± 2.0% (Figure 4E). This resulted in a calculated CFU reduction of 88.4 ± 2.7%, which could not be further increased with 200 µM TMPyP (reduced by 90.5 ± 1.1%, Figure 4F). This result correlated well with the counted bacterial density, which was reduced by 92.5 ± 8.6% under 120 min illumination without TMPyP (*r* = 0.719; *p* < 0.0001, Figure 4G) and reduced by 99.8 ± 0.2% with 200 µM TMPyP (Figure 4H). Again, the effects of aPDI were underestimated with the luminometric method.

For VRE *Enterococcus faecium*, which has previously shown a high susceptibility to TMPyP treatment already at the lowest concentration investigated (50 µM, 60 min illumination), we observed a reduction in luminescence of 93.1 ± 1.9% (Figure 4I). Here, our calculation method resulted in a bacterial density less than zero (Figure 4J). These findings indicate an increased scattering between calculated and manually detected values and a lower sensitivity of the luminescence method at low bacterial densities (*r* = 0.236, *n* = 38, *p* = 0.1531, Figure 4K). The susceptibility of VRE *Enterococcus faecium* to TMPyP-based aPDI was confirmed by manually determined bacterial density (reduction of 99.99 ± 0.02%; Figure 4L), thereby confirming the finding of our previous study [34].

In addition to the pronounced aPDI effect, a dark toxicity of TMPyP was observed in VRE *E. faecium*. To this end, the luminescence detected in samples that were exposed to 200 µM TMPyP but kept in the dark was already reduced by 80.6 ± 3.5% (Figure 4I). To further examine the observed dark toxicity of TMPyP in *E. faecium*, the ATP-induced luminescence was detected before and after incubation with TMPyP (200 µM) for 120 min in the dark. In samples treated with TMPyP, the luminescence increased from 11.7 × 10^4^ ± 0.8 × 10^4^ RLU to 35.5 × 10^4^ ± 4.0 × 10^4^ RLU, whereas the ATP-induced luminescence of untreated samples increased from 11.2 × 10^4^ ± 0.8 × 10^4^ to 66.0 × 10^4^ ± 0.3 × 10^4^ RLU (Figure S2B).

Consistent with previous data, MDR *Pseudomonas aeruginosa* revealed a significant background luminescence most likely caused by a pyoverdine-induced luminescence [35] that increases the luminescence of bacteria (Figure 4M). This increased background luminescence hampers the calculation of bacterial density. Consequently, the correlation of the calculated and actually observed density of the bacteria was very poor, especially at lower bacterial densities (*r* = 0.443, *n* = 12, *p* = 0.1488). Calculated values less than zero were excluded from the calculations (Figure 4O). The increased cut-off precluded a distinction of aPDI effects at different TMPyP concentrations (Figure 4N,O). Therefore, bioluminescence of bacterial strains seems to be an exclusion criterion for this method. However, we observed a reduction in bacterial density of 99.8 ± 0.27% after illumination with blue light without a photosensitizer. With TMPyP-based aPDI, we observed a concentration-dependent effect of up to a complete inactivation at 200 µM TMPyP followed by 120 min illumination, confirmed with manual bacteria counting (Figure 4P).

In the following TMPyP-based aPDI experiments with different bacterial strains, we observed an acceptable correlation between the luminescence-based measurements and manual colony counting: Carbapenem-resistant Enterobacteriaceae (CRE) *Klebsiella pneumoniae* (*r* = 0.706; *p* < 0.0001; Figure S3A), MRSA *Staphylococcus aureus* (*r* = 0.894; *p* < 0.0001; Figure S3B), XDR *Achromobacter xylosoxidans* (*r* = 0.696; *p* < 0.0001; Figure S3C). The corresponding data acquired after aPDI with luminescence measurements and manually determined bacterial densities are presented in Figure S3.

### 3.5. Using the New Protocol for Characterization of aPDI Effects Elicited by Another Photosensitizer

**Figure 5:**
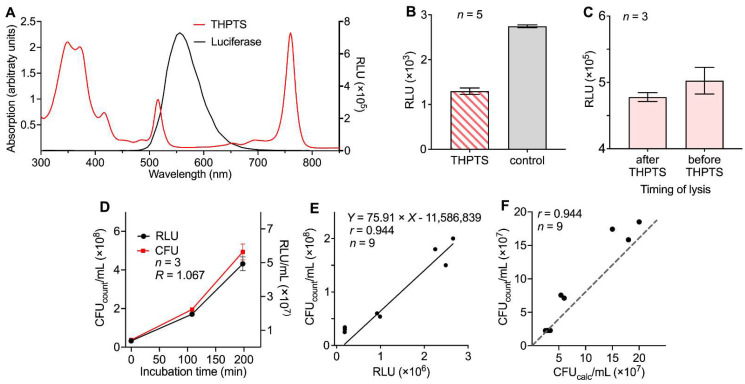
Correlation of bacterial densities as determined by luminescence and manually counting in the presence of the photosensitizer THPTS: (**A**) The absorption spectrum of THPTS dissolved in 0.9% NaCl was measured. Additionally, the luminescence spectrum of the luciferase used in the cell viability assay is presented. (**B**) Background luminescence of 50% BHI broad medium (dissolved in 0.9% NaCl) with 200 µM THPTS compared to 50% BHI broad medium alone. (**C**) Bacteria-lysing property of the assay was measured with and without THPTS in *M. morganii*. (**D**) Bacterial growth curve of VRE *E. faecium*, incubated in BHI broad medium at 37 °C, 5% CO_2_, detected at indicated time points. Density of bacteria was determined by luminometric assay and colony counting (CFU_count_). (**E**) Correlation between results from luminometric readings and calculated colony density (CFU_calc_) in VRE *E. faecium*, with results of luminometry in heat-inactivated bacteria used to define zero CFU/mL. (**F**) Correlation between CFU_calc_ and CFU_count_ of VRE *E. faecium; r* = correlation coefficient.

To transfer the protocol for the characterization of aPDI effects to a different photosensitizer, we selected THPTS, which has previously been shown to have significant clinical potential as a photosensitizer that can be activated by near-infrared light. The initial calibration experiments were performed as described above (Figure 3). THPTS has several absorption peaks with a maximum at λ = 760 nm, which is used during aPDI treatment for excitation, but also a further absorption maximum at λ = 516 nm that partially overlaps with the luminescence spectrum of the luciferase used in our assay (Figure 5A). Evaluation of the photosensitizer-caused quenching factor revealed a reduction in the luciferase-caused luminescence of 52.8 ± 4.9% in the presence of 200 µM THPTS in BHI broad medium (Figure 5B). As described above for the TMPyP experiments, the lysing property of the assay was examined with and without THPTS in *M. morganii* bacteria. No relevant influence of THPTS on lysing effectivity was observed (Figure 5C).

When performing THPTS-based aPDI experiments, a similar reduction in luminescence and manually determined bacterial density was found in VRE *E. faecium* (Figure 5D). As described above for TMPyP, the bacterial growth rate (R) between 108 min and 198 min (corresponds to 90 min incubation after a typical aPDI experiment with THPTS) was established (Figure 5D), and the ATP-elicited luminescence was determined in samples from the corresponding bacterial lysates in the presence of THPTS. Together with data from the manual bacterial density determination (*t* = 0, 108, 198 min), a linear regression analysis for the luminescence data was performed. This analysis indicated a good correlation between the data obtained from the luminometric measurements and the observed bacterial density as assessed by classical clonogenic CFU determination in THPTS-based aPDI experiments (*r* = 0.944, *p* < 0.0001, Figure 5E). Taken together, these data demonstrate that luminometric data in the presence of THPTS could be used to calculate bacterial density with acceptable accuracy (Figure 5F).

**Figure 6:**
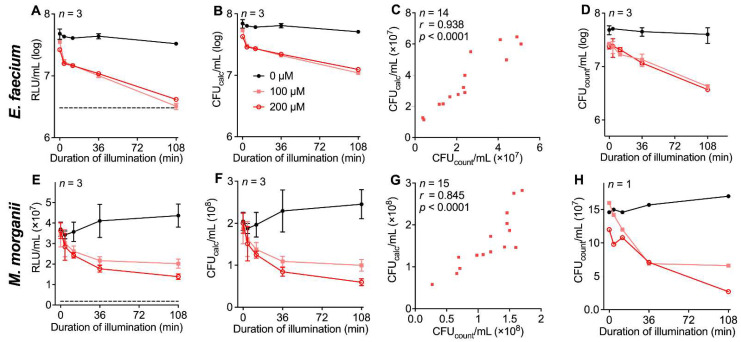
Correlation of calculated and manually detected bacterial density for THPTS-based aPDI in different bacterial types. THPTS was illuminated with 18 mW/cm^2^ resulting in 0 J/cm^2^ (0 min), 4.3 J/cm^2^ (4 min), 12.9 J/cm^2^ (12 min), 38.9 J/cm^2^ (36 min), 116.6 J/cm^2^ (108 min) at λ_cent_ =760 nm. Dashed line indicates the luminescence of heat-inactivated bacteria. (**A**) ATP-induced luminescence by VRE *E. faecium* after aPDI. (**B**) Calculated bacterial density of VRE *E. faecium* based on luminometric measurements. (**C**) Correlation between calculated and manually determined bacterial density in VRE *E. faecium*; r = correlation coefficient. (**D**) Manually counted bacterial density of VRE *E. faecium* after aPDI. (**E**–**H**) Results for *M. morganii* as described above for *E. faecium*.

To identify the aPDI effects of THPTS on VRE *E. faecium* and *M. morganii*, the red-light susceptibility or dark toxicity of THPTS was determined. For both bacterial strains, no effect from red light or the photosensitizer alone were observed. VRE *E. faecium* offered, after aPDI, a THPTS concentration-dependent decrease in luminescence, with the greatest reduction of 91.1 ± 1.5% RLU (200 µM THPTS; 108 min illumination; Figure 6A). This corresponded to a decreased bacterial density by 81.7 ± 2.5% CFU calc/mL (Figure 6B). When comparing all calculated and manually counted values for the density of viable bacteria, we observed a correlation of *r* = 0.938 (*p* < 0.0001, Figure 6C). Moreover, analyzing the manually determined values, we found a similar magnitude of density reduction of 92.20 ± 1.09% (Figure 6D).

For *M. morganii*, we observed a reduction in ATP-induced luminescence of 62.5 ± 0.4% RLU after THPTS-based aPDI (Figure 6E), which resulted in a calculated reduction in bacterial density of 65.6 ± 0.4% (Figure 6F). Again, there was an acceptable correlation between the calculated and manually observed values for bacterial density (*r* = 0.845; Figure 6G,H).

## 4. Discussion

To establish a high-throughput method to assess the effects of various aPDI treatments on a great variety of different bacteria, we evaluated the feasibility and limitations of a luminescence-based method, which detects bacterial ATP as a surrogate of bacterial survival.

Although a direct use of the protocols provided by the manufacturer resulted in significant differences in the effect size as determined by the luciferase-based approach and conventional colony counting after preparation of bacterial cultures on agar plates, we were able to establish a protocol that allows for the standardized testing and calibration of the luminescence-based bacterial density measurements and the subsequent comparison of obtained results with previously obtained data from conventional bacterial colony counting.

The feasibility of this approach was shown to depend, on the one hand, on the bacterial strain used, and on the other hand, on the optical properties of the PS. We found that the investigated luminescence-based method is feasible in many of the bacterial strains tested, and the aPDI effects determined by this approach were in good agreement with those obtained by manual counting of bacterial colonies after agar plate culturing [32]. In some bacterial strains, such as MDR *Pseudomonas aeruginosa*, however, the intrinsic properties of the bacteria precluded the use of luminescence-based cell density determination. For other bacterial strains, e.g., *E. coli*, our results demonstrate that a universal conversion of the results obtained by both methods is also not possible. In contrast, individual calibrations are required to account for different types of bacteria, growth media and photosensitizers and, thereby, to minimize the margin of error.

After these initial calibration experiments, the luminescence-based analysis of bacterial viability allowed us to analyze the effects of aPDI with shorter processing times and increased scalability, as compared to the conventional procedure of bacterial cultures and manual colony counting.

Although we considered the clonogenic (growth) method (manual colony counting) as the gold standard for bacterial density detection, we want to emphasize that this method does not allow for the detection of effects that do not lead to a reduced number of colonies but result, e.g., in a slower metabolism, as described by Kramer et al. [36], or in a reduced ability to replicate, which may be caused by aPDI-induced photooxidative DNA damage [37]. Therefore, ATP-based methods may reveal additional facets of the bacteriostatic effects of aPDI [36]. In support of this hypothesis and by using the luminescence method as described here, we could identify a dark toxicity of TMPyP in *E. faecium* that is caused not by a bactericidal but by a bacteriostatic effect, which was not apparent from the conventional colony-counting method. As TMPyP is a positively charged quaternary amine, some antibacterial properties may be expected at concentrations used in our aPDI experiments [38,39,40,41].

Using this novel luminescence approach, we could demonstrate the different susceptibilities of several bacterial strains to aPDI. Although aPDI is considered to exert its bactericidal and/or bacteriostatic effect via the generation of ROS, which should affect all bacteria, these variations may result from differences in the accumulation of photosensitizers at the bacterial plasma membrane and other targets of aPDI-induced ROS. Additionally, diverging activities of the repair mechanisms [15,42,43] for ROS-induced damages and/or the systems that mediate the efflux of photosensitizers for the bacteria [44] may contribute to the observed differences in aPDI sensitivity.

Interestingly, in most experiments, the aPDI effect was underestimated by the here-described luminescence-based method as compared to results from colony counting. This may result from a systematic error, since the ATP concentration determined in heat-inactivated bacterial samples at the timepoint *t* = 0 min was defined as the baseline ATP concentration corresponding to a bacterial density of zero in manual counting. In the corresponding aPDI treated samples, the bacteria may still produce significant amounts of ATP before being eliminated by aPDI treatment. It is well-known that photodynamic treatment can change the growing conditions and, subsequently, the ATP synthesis of different bacterial strains in a not-negligible way [36]. Nevertheless, as observed with the high susceptibility of *E. faecium* to TMPyP-based aPDI, the complete inactivation of bacteria could also be verified by the luminometric method. In this bacteria strain, aPDI apparently reduced the ATP concentration to a greater extent than heat inactivation did, which led to the observed negative values for bacterial density as a result of the predefined cut-off based on heat inactivation.

Another critical factor when applying the luminescence method evaluated here is the optimal duration of the post-incubation time of bacterial samples before luminescence detection. Here, optimal results will require an individual testing for each type of bacteria, growing condition and PS. An optimized post-incubation duration may increase the correlation between manual counting and the proposed luminometric method.

Our experiments demonstrated that the optical properties of the PS, as well as the growth media used, may influence the detection of luminescence due to their absorption characteristics. For most components, such as the BHI, this effect was constant and did not change during illumination and could therefore be easily compensated. Absorption properties of other growth media, e.g., blood-containing solutions as well as of some PS, however, may change during aPDI treatment and therefore contribute to the underestimation of aPDI effectivity with the ATP-based luminescence method.

Using the PS THPTS, we tested whether the measurement of the ATP concentration by luminescence detection is suitable to determine the aPDI effect of a given combination of PS and bacteria. Our results indicate that it is possible in most cases to detect the dose-dependent effects of aPDI in detail and for large sample sizes. Combinations that are not suitable for this method, however, must be identified in initial tests beforehand. Moreover, it is necessary to adjust the protocol to specific bacteria and photosensitizers to allow for the comparability of the results obtained by the luminescence method and conventional colony counting. Some bacterial strains, such as MDR *Pseudomonas aeruginosa*, are not suitable for this approach, in this specific case due to auto-luminescence [35].

Although we could demonstrate that the luminescence method described here is suitable to determine aPDI effects in vitro, we cannot judge on its feasibility in samples obtained from in vivo aPDI treatments. Here, more adjustments to this method may be required to account for the influence of additional preanalytical confounders, such as the unknown photosensitizer concentration and other contaminants in the samples.

At the time of writing, an ATP-based approach as described here requires assay reagents and disposables of approximately USD 0.25 per sample (not considering probable cost reductions by scaling effects). While the costs of consumables for the classical clonogenic method (preparation of bacterial cultures) may be lower, one should take into account that higher labor costs are likely to arise, at least if manual culture preparation and colony counting are performed.

Based on data from previous studies, the illumination at the indicated doses should be well-tolerated by eukaryotic cells [45,46,47]. However, our in vitro study cannot rule out adverse effects of aPDI in adjacent host tissues.

In conclusion, the evaluated luminescence method is an interesting alternative for the in vitro investigation of aPDI effects in many bacterial strains—especially if a high number of samples are to be processed simultaneously, e.g., in order to explore the dose–response relationships of a novel PS or to determine the optimal incubation and illumination times in various bacterial strains. Here, initial calibration experiments are still required to allow for a good agreement of the obtained results to those from the clonogenic method. While the clonogenic method has its own limitations, e.g., strains of bacteria that are difficult to cultivate, it is certainly more robust with regard to the interference of photosensitizers, growth medium and bacterial autofluorescence. Despite these limitations, the measurement of bacterial density by ATP-dependent luminescence provides the basis for a scalable multidimensional approach to assess the aPDI effects of a given bacteria/PS combination.

## Data Availability

Data are contained within the article or Supplementary Material.

## Supplementary Materials

The following supporting information can be downloaded at: https://www.mdpi.com/article/10.3390/microorganisms10050950/s1, Figure S1: Luminescence of heat-inactivated bacteria compared to active bacteria, dependent on post-incubation time, Figure S2: Background luminescence of bacteria growth media and VRE *E. faecium* susceptibility to TMPyP without illumination, Figure S3: Further evaluation of aPDI effects of TMPyP on different bacterial strains as determined by ATP-evoked luminescence and manual counting of bacteria colonies.
