# Supplementary material for: Evaluation of a Luminometric Cell Counting System in Context of Antimicrobial Photodynamic Inactivation
Source: Microorganisms. 2022 Apr 30;10(5):950. doi: 10.3390/microorganisms10050950 (PMC9147394; doi:10.3390/microorganisms10050950)
Supplement: Supplementary file 1 [file microorganisms-10-00950-s001.zip › microorganisms-1683188-supplementary.pdf]

microorganisms

MDPI

Supplemental materials

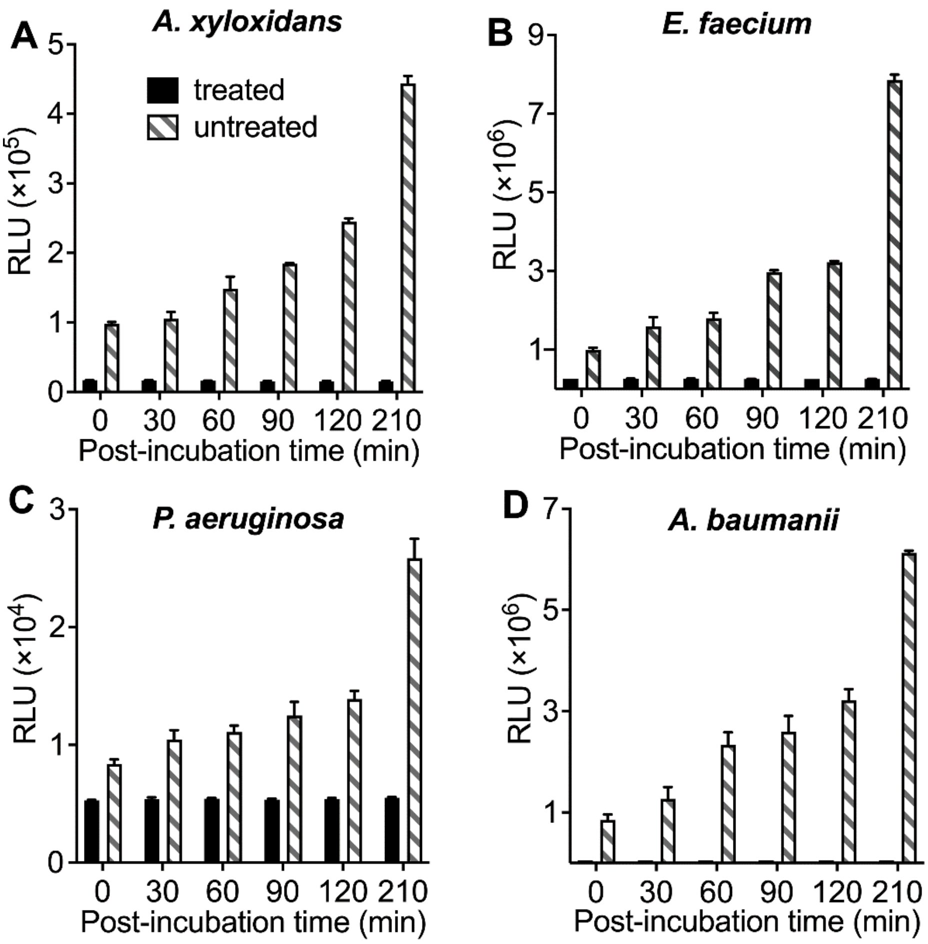

Figure S1. Luminescence of heat-inactivated bacteria compared to active bacteria, dependent on post-incubation time. Bacteria suspended in 50 % BHI broad medium were heat-inactivated or left untreated and incubated at 37 °C, 5% CO_2. The ATP-evoked luminescence was measured on bacterial lysates with the cell viability assay at the indicated timepoints in **(A)** XDR *Achromobacter xylosoxidans*, **(B)** VRE *Enterococcus faecium*, **(C)** MDR *Pseudomonas aeruginosa and* **(D)** XDR *Actinetobacter baumanii* (n = 3 each).

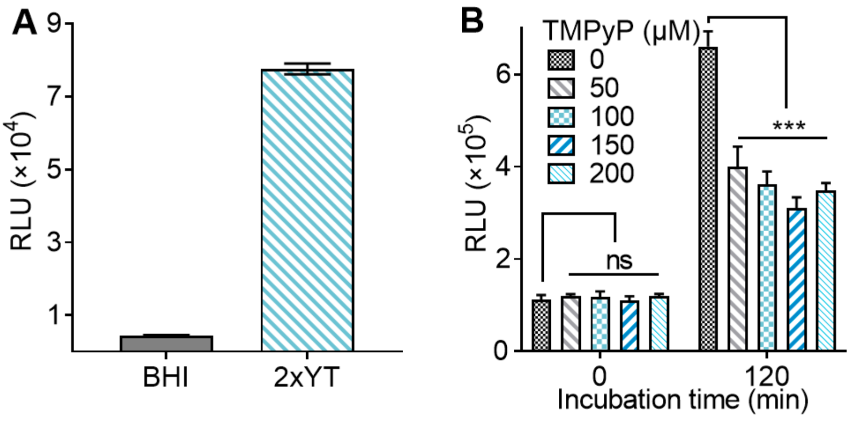

Figure S2. Background luminescence of bacteria growth media and VRE *E. faecium* susceptibility to TMPyP without illumination (**A**) Background luminescence varies between bacterial growth media. The BHI growth medium has a 17 times lower luminescence compared to 2xYT growth medium, when measured without photosensitizer ($n = 8$). **(B)** ATP-induced luminescence in VRE *E. faecium* after 0 min and 120 min incubation with TMPyP without illumination at 37 °C, 5% CO_2 (*** $p < 0.001$, two-way ANOVA and Bonferroni correction, $n = 3$).

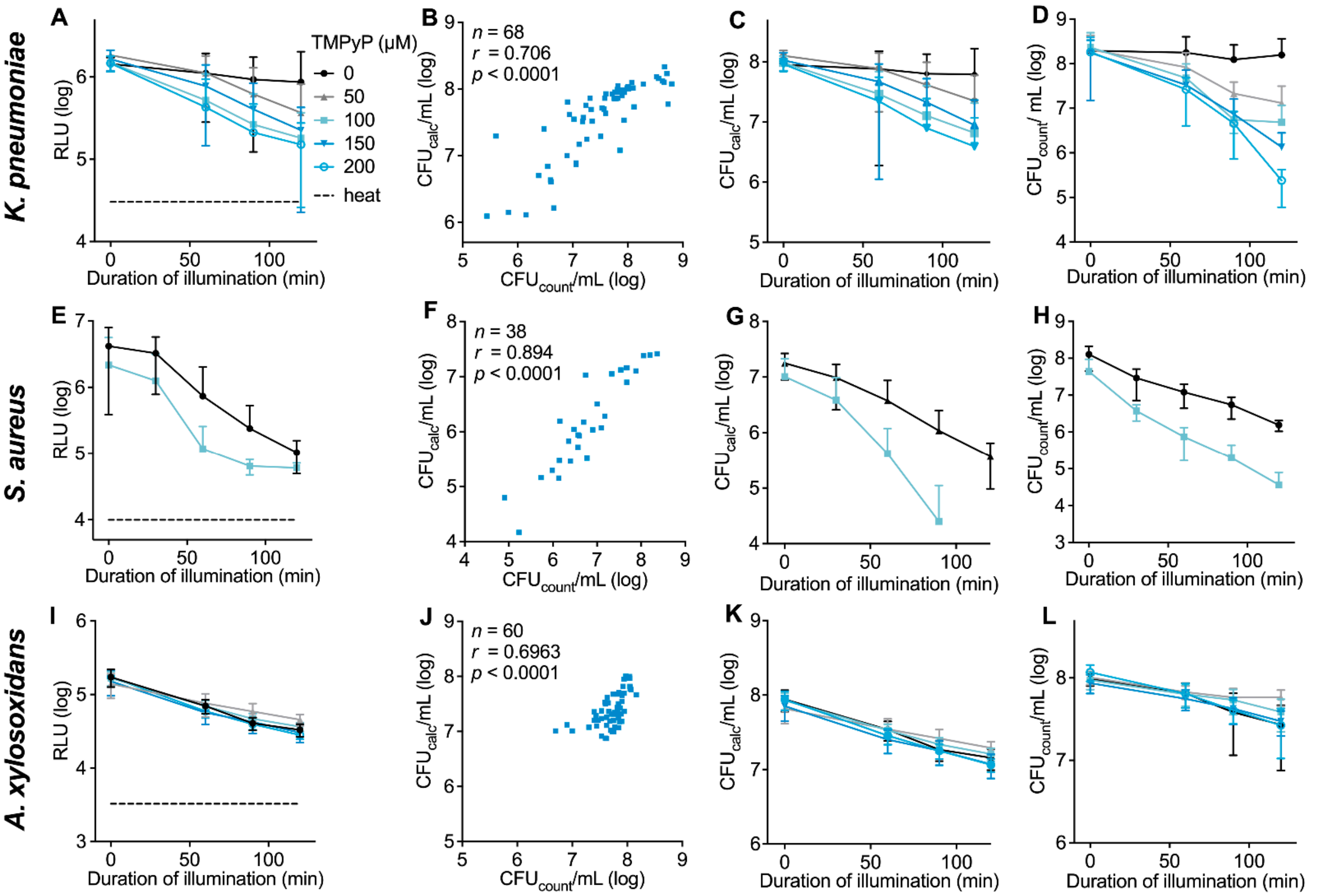

Figure S3. Further evaluation of PDI effects of TMPyP on different bacterial strain as determined by ATP-evoked luminescence and manual counting of bacteria colonies. Data from PDI experiments are presented. TMPyP was illuminated with 13 mW/cm^2 results in 0 J/cm^2 (0 min) 46.8 J/cm^2 (60 min), 70.2 J/cm^2 (90 min), 93.6 J/cm^2 (120 min) at $\lambda_{cent} = 420$ nm, dashed line shows the luminescence of heat-inactivated bacteria. (**A**) ATP-induced luminescence by CRE *K. pneumoniae*

after PDI. (**B**) Correlation between calculated (CFU_{calc})and manually determined bacterial density (CFU_{count})in CRE *K. pneumoniae*; r = correlation coefficient. (**C**) CFU_{calc} of CRE *K. pneumoniae* based on luminometric measurements. (**D**) CFU_{count} of CRE *K. pneumoniae* after PDI. (**E-H**) Results for MRSA *S. aureus* as described above for *K. pneumoniae*. (**I-L**) Results for XDR *A. xylosoxidans* as described above for *K. pneumoniae*.
